# Nanoparticle technologies for liver targeting and their applications in liver diseases

**DOI:** 10.3389/fbioe.2025.1661872

**Published:** 2025-10-23

**Authors:** Mengjia Peng, Fei Fang, Bowen Wang

**Affiliations:** ^1^ Department of Emergency, The General Hospital of Tibet Military Command, Lhasa, China; ^2^ Department of Gastroenterology, Laboratory of Gastroenterology and Hepatology, West China Hospital, Sichuan University, Chengdu, China; ^3^ State Key Laboratory of Oral Diseases, National Center for Stomatology, National Clinical Research Center for Oral Diseases, West China Hospital of Stomatology, Sichuan University, Chengdu, China

**Keywords:** nanoparticles, hepatic cells, hepatic sinusoids, liver targeting, liver diseases

## Abstract

Liver diseases represent a significant global health challenge, affecting millions of lives annually. The advent of nanoparticle (NP) technologies has introduced promising therapeutic strategies for addressing liver diseases. Given the liver’s pivotal role in detoxification and the inherent ability to interact with circulating NPs, it emerges as an ideal target for NP-mediated therapies. Upon systemic administration, NPs predominantly accumulate within the liver, where they are uptaken and internalized by hepatic macrophages, sinusoidal endothelial cells, and hepatocytes. This natural tropism of NPs toward the liver highlights their potential for targeted liver disease management. This review describes the physiological conditions of the hepatic sinusoids and elucidates the interactions between various hepatic cells and NPs. A thorough understanding of these physiological mechanisms is essential for optimizing liver-targeted NP delivery systems, thereby improving NP accumulation at pathological sites. The development of liver-targeted NPs technologies holds immense promise for both the diagnosis and treatment of liver diseases.

## 1 Introduction

Liver diseases are widely prevalent all over the world, affecting individuals in both low-income countries and high-income countries (Gines et al., 2021; Adebayo et al., 2019). Annually, approximately two million lives are lost due to liver related diseases, one million attributed to cirrhosis, and one million attributed to viral hepatitis and hepatocellular carcinoma (HCC) (Yeo et al., 2024; Do et al., 2024). The spectrum of liver diseases encompasses acute liver failure, various forms of hepatitis (viral, alcoholic, fatty, metabolic), cirrhosis, and HCC (Targher et al., 2024). These conditions not only inflict direct damage upon the liver parenchyma but also disrupt hepatic metabolism of carbohydrates, lipids, and proteins, leading to systemic metabolic derangements characteristic of liver disease patients (Walradt and Jirapinyo, 2024). Consequently, the impaired hepatic function significantly hampers the uptake and utilization of numerous drugs, posing substantial challenges to the development of effective liver-targeted therapies (Ngo et al., 2022).

In recent years, nanoparticle (NP) technologies have emerged as a groundbreaking frontier in medical research, demonstrating remarkable progress across diverse therapeutic domains (Lan et al., 2024; Li S. et al., 2024). NPs delivery systems hold the potential to revolutionize drug distribution within the body by prolonging systemic circulation times and facilitating targeted delivery to pathological sites (Yang L. et al., 2024). NPs encompasses five groups based on the nanoconstructs, including inorganic metal NPs, carbon-based NPs, lipid NPs, polymeric NPs, and nucleic acid NPs (Zhang JA. et al., 2024). Through strategic modifications, these NPs can be tailored for organ-specific targeting, thereby enhancing therapeutic efficacy while minimizing off-target effects. The escalating demand for advanced therapies has propelled several NP formulations into clinical trials, heralding a new era in precision medicine (Mitchell et al., 2021; Sayour et al., 2024).

NPs hepatic uptake is achieved through passive or active means. Passive uptake is non-specific, primarily mediated by the mononuclear phagocyte system (MPS) capturing unmodified particles. Active targeting enhances specificity by decorating nanomaterials with targeting moieties (e.g., antibodies, peptides) that bind to unique receptors on particular liver cells, such as hepatocytes, reducing off-target sequestration and improving delivery efficiency (Bottger et al., 2020). However, a significant limitation of NP-based therapies lies in their rapid clearance by MPS, which constitutes a major barrier to effective drug delivery (Hulugalla et al., 2024). The MPS, primarily composed of macrophages residing in the liver (Kupffer cells) and spleen, functions as the body’s filtration system, actively sequestering and internalizing circulating NPs (Zelepukin et al., 2024). Studies indicate that nearly 85% of liver macrophages and 25% of splenic macrophages will accumulate NPs, underscoring the liver’s pivotal role in NP biodistribution (Tsoi et al., 2016). Meanwhile, this phenomenon also positions the liver as an optimal target organ for nanotherapeutics, given its inherent capacity to accumulate NPs. By integrating passive hepatic uptake mechanisms with active targeting strategies, the therapeutic potential of NPs in managing liver diseases can be substantially augmented (Liu et al., 2024).

This review aims to provide a comprehensive overview of NP-mediated targeting strategies and their applications in the treatment of liver diseases. We commence by elucidating the microanatomical features of the liver and the implications of hepatic sinusoidal architecture on NP accumulation and clearance. Subsequently, we delve into the impact of NP characteristics on their biodistribution and liver-targeting efficiency. Lastly, we highlight the therapeutic prospects of NPs in addressing acute liver failure (ALF), non-alcoholic fatty liver disease (NAFLD), liver fibrosis, and HCC, emphasizing the transformative potential of nanotechnology in advancing liver disease management.

## 2 The accumulation of NPs in the liver

### 2.1 Hepatic sinusoidal architecture makes liver an ideal organ for NPs accumulation

The liver, being one of the most vital organs in the human body, plays a crucial role in metabolic processes, detoxification, and protein synthesis. Liver receives a substantial blood supply, accounting for approximately 1.5 L per minute, which is among the highest perfusion rates in the body (Kan et al., 2008; Seifalian et al., 1991). This blood is delivered through two primary vessels: the hepatic artery and the portal vein, contributing 30% and 70% of the total hepatic blood flow, respectively (Abdel-Misih and Bloomston, 2010). The hepatic artery supplies oxygenated blood, while the portal vein delivers nutrient-rich blood from the gastrointestinal tract. Within the hepatic sinusoids, blood from these vessels mixes, undergoes metabolic exchange, and is subsequently drained via the central veins. Notably, despite the rapid flow in the afferent vessels, the blood velocity within the sinusoids dramatically decreases to 1/1000th that of the portal vein, creating a low-velocity environment conducive to NP adhesion and retention (Tsoi et al., 2016).

**FIGURE 1:**
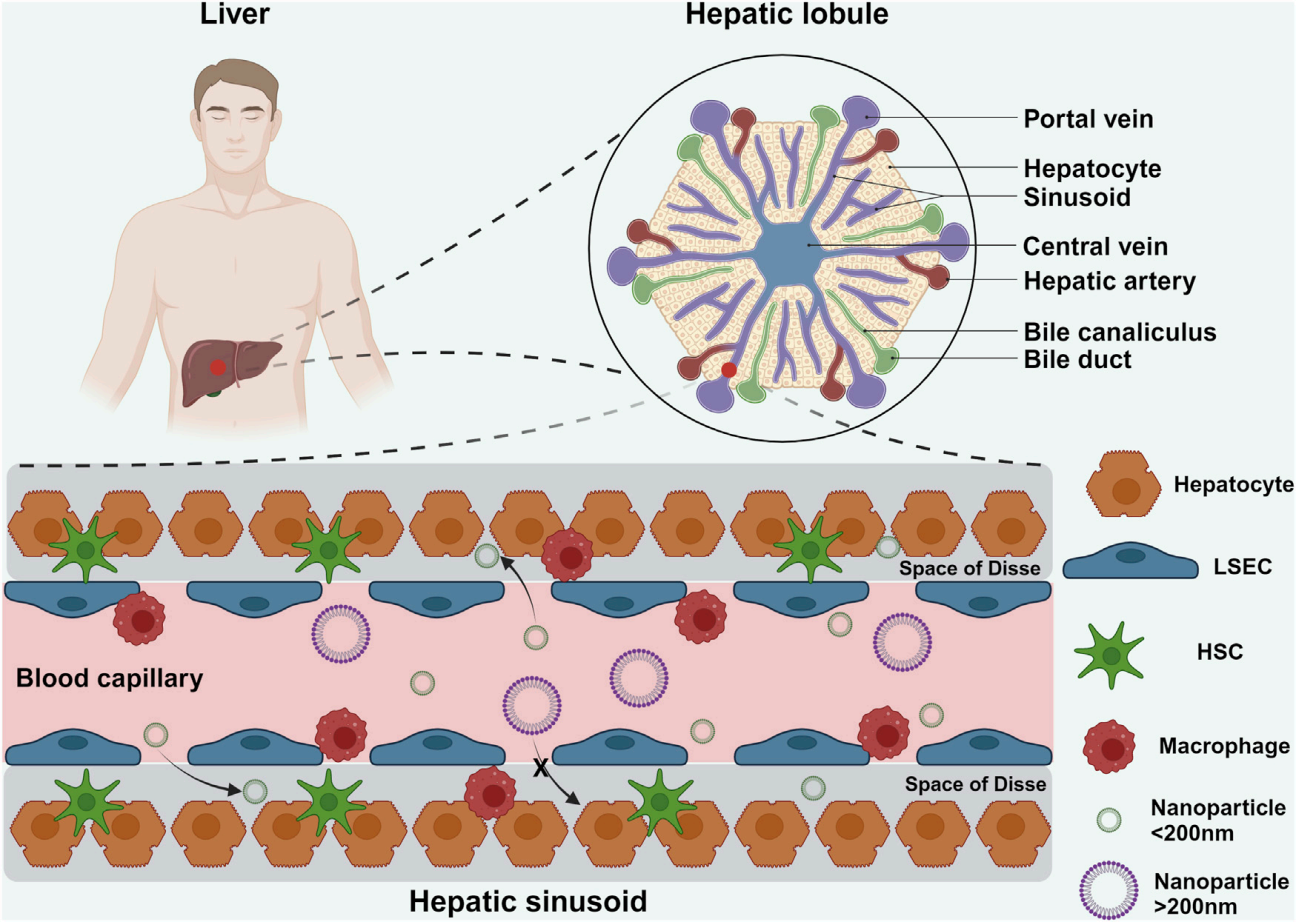
Schematic illustration showing the hepatic lobule and hepatic sinusoid. The hepatic lobule represents the smallest structural and functional unit of the liver. Within the hepatic lobule, blood from the hepatic artery and portal vein mixes in the sinusoids before draining into the central vein. LSECs lining the sinusoids are closely arranged along the inner vessel wall, with intercellular gaps ranging from 50 nm to 200 nm. NPs smaller than 200 nm can traverse these endothelial gaps to interact with HSCs and hepatocytes within the liver sinusoids (Feng and Gao, 2021). Created in https://BioRender.com.

At the cellular level, the liver comprises parenchymal cells (hepatocytes, constituting 60% of all liver cells) and non-parenchymal cells (40% of all liver cells), including liver sinusoidal endothelial cells (LSECs), hepatic stellate cells (HSCs), and Kupffer cells (KCs) (Figure 1) (Feng and Gao, 2021). Hepatocytes are organized in single-cell cords towards the center of the lobule, making contact with the sinusoidal blood vessels (Gao, 2016). LSECs and hepatocytes are separated by a region known as the space of Disse. These spaces facilitate the exchange of small molecules and nutrients between blood and hepatocytes. LSECs form a fenestrated barrier with pores ranging from 50 nm to 200 nm in diameter, functioning akin to a selective filter that promotes efficient uptake of substances by hepatocytes (Gao, 2016). HSCs, rich in lipid droplets and vitamin A, contribute to the extracellular matrix formation. KCs, dispersed among LSECs, act as the liver’s resident macrophages, engulfing foreign particles and debris from the bloodstream (Wang et al., 2021).

When NPs enter the hepatic sinusoids, their interaction with this intricate microenvironment significantly influences their biodistribution and fate. Due to the slow blood flow, a substantial portion of NPs adheres to the LSECs, facilitating their uptake by KCs (Lankveld et al., 2010). However, not all NPs are cleared in this manner; a fraction evades phagocytic surveillance and is internalized by LSECs. NPs larger than 200 nm are shunted into the systemic circulation and eventually sequestered by the spleen, whereas smaller NPs (<200 nm) penetrate deeper into the liver parenchyma, interacting directly with hepatocytes and HSCs (Lankveld et al., 2010; Boey and Ho, 2020).

### 2.2 Route of NPs administration promotes the accumulation of NPs in the liver

The route of administration plays a crucial role in determining the biodistribution and accumulation of NPs within the body, with the liver being a primary target organ for NPs accumulation due to its extensive blood supply and unique sinusoidal architecture (Xu et al., 2023). The most common routes of administration in clinical practice and animal models include oral, intravenous, intramuscular, and intraperitoneal injections (Bitounis et al., 2024). Each route has distinct advantages and challenges that influence the efficiency of NPs delivery and subsequent hepatic accumulation.

Oral administration is often preferred due to its convenience and high patient compliance. However, this route presents significant challenges as NPs must navigate the complex gastrointestinal environment (Shen et al., 2024; Ramire et al., 2024). Gastric juices, pancreatic enzymes, intestinal fluids, and bile can all contribute to the unexpected loss of NPs (Barros et al., 2024). Additionally, the penetrability of NPs through the mucus layer and intestinal epithelium is vital for their absorption and bioavailability (Xu et al., 2024). Furthermore, the gastrointestinal tract is rich in macrophages and dendritic cells capable of clearing substantial quantities of NPs (Tranah et al., 2021; Pabst et al., 2023). Despite these obstacles, an adequate dose of NPs can evade these defenses and enter systemic circulation.

In contrast, intravenous, intramuscular, and intraperitoneal injections bypass the digestive tract, thereby overcoming some of the barriers associated with oral administration and potentially enhancing bioavailability. Intravenous injection provides the most direct and highest initial hepatic exposure, as NPs enter the systemic circulation directly, bypassing other absorption barriers and allowing immediate interaction with liver sinusoids and Kupffer cells. However, these routes may suffer from reduced practicality due to factors such as invasiveness, potential for localized side effects, and the need for specialized administration procedures (Wang et al., 2023).

Regardless of the administration route, NPs ultimately enter systemic circulation. Venous blood from the head, neck, and upper limbs returns to the heart before entering the portal system. Blood from the lower limbs enters the systemic circulation via the inferior vena cava (Wang et al., 2023; Kulchar et al., 2023). Given the liver’s rich blood supply, NPs inevitably pass through the liver where they undergo metabolism. This physiological process highlights the liver’s central role in NP clearance and underscores the importance of understanding how different administration routes impact NP biodistribution and hepatic accumulation.

### 2.3 The interactions of NPs with various hepatic cells in the liver disease

The innovative NPs are designed to interact specifically with the diverse cellular populations of the liver—including KCs, HSCs, LSECs, and hepatocytes—each playing distinct roles in disease progression and treatment response. Several NPs-based formulations have already progressed to clinical trials, demonstrating promising potential for enhancing drug delivery, improving therapeutic efficacy, and reducing systemic side effects (Table 1). This section summarizes the mechanisms through which NPs interact with different hepatic cell types in both healthy and diseased microenvironments, with particular emphasis on those systems that have reached clinical-stage development, thereby bridging foundational research with translational applications.

**TABLE 1:** Nanoparticle systems for liver disease in clinical trials or commercialized.

Nanoparticle systems	NPs formulation	Delivered drugs	Stage	References
Lipid-based NPs	VA-liposomes	Imatinib	Phase Ⅱ	Krischer et al. (2017)
Lipid-based NPs	VA-liposomes	Valsartan	Phase Ⅱ	Easa et al. (2019)
Lipid-based NPs	pPB-modified liposomes	Recombinant human TRAIL	Phase Ⅲ	Li et al. (2019)
Polymer-based NPs	Cationic nanohydrogel particles	siRNA	Phase Ⅲ	Duran-Lobato et al. (2021)
Polymer-based NPs	Ketal cross-linked cationic nanohydrogel	Anti-col1α1 siRNA	Phase Ⅱ	Leber et al. (2017)
Polymer-based NPs	PLGA	phyllanthin	Phase Ⅱ	Zhang et al. (2018)
Polymer-based NPs	PEG-PLGA/PLGA NPs	sorafenib	Phase Ⅱ	Lin et al. (2016)
Inorganic NPs	Mesoporous silica NPs	siTnC	Phase Ⅲ	He et al. (2010)
Inorganic NPs	PEG-AuNPs	hesperetin	Phase Ⅱ	Vivero-Escoto et al. (2019)
Inorganic NPs	AuNPs and SiNPs	NO donors	Phase Ⅱ	Krishnan et al. (2017)
Inorganic NPs	PtNPs	Curcumin	Phase Ⅱ	Das et al. (2010)
Inorganic NPs	Calcium phosphate NPs (CaP@BSA NPs)	TSG-6	Phase Ⅱ	Wang et al. (2020)

**FIGURE 2:**
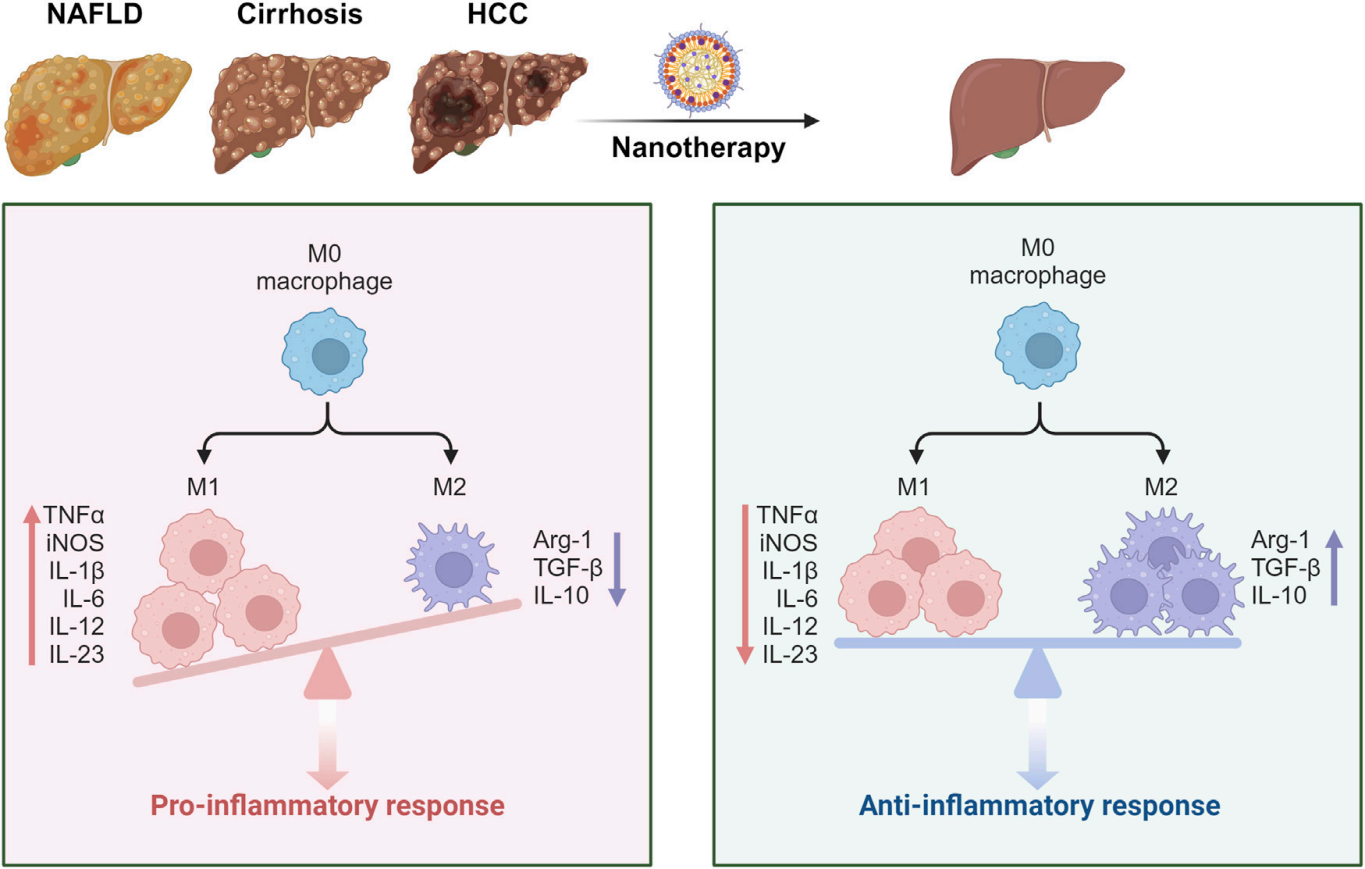
Schematic illustration showing the NPs targeting the macrophages to alleviate liver diseases. In the context of liver disease, KCs polarize towards the M1 phenotype, exacerbating hepatic inflammation through the secretion of pro-inflammatory cytokines. Following NP treatment, there is a shift in KC polarization towards the M2 phenotype, which alleviates inflammation by secreting anti-inflammatory cytokines. This transition ultimately contributes to the amelioration of liver disease (Zhang W. et al., 2024; Hu Y. et al., 2024). Created in https://BioRender.com.

Upon entering the hepatic sinusoids, NPs first encounter KCs, which constitute a vital component of the MPS, accounting for approximately 80% of the body’s macrophages (Barreby et al., 2022). KCs play an essential role in liver immune regulation and maintaining immunological homeostasis. KCs are categorized into two distinct subpopulations based on their functional roles and secretion profiles: M1 and M2 macrophages (Wang Y. et al., 2024). M1 macrophages, activated by lipopolyscharide (LPS) and interferon-gamma (IFN-γ), secrete high levels of interleukin-2 (IL-2) and lower levels of interleukin-10 (IL-10), primarily promoting inflammation, bactericidal activity, and phagocytosis (Wang R. et al., 2024). Conversely, M2 macrophages, activated by interleukin-4 (IL-4), predominantly secrete anti-inflammatory cytokines such as IL-10, thereby suppressing local inflammatory responses (Hou et al., 2024). Numerous studies have demonstrated that NPs can facilitate the conversion of M1 to M2 macrophages through drug delivery, mitigating macrophage-driven inflammatory responses and thus alleviating liver disease (Figure 2) (Jain et al., 2024; Zhang W. et al., 2024; Hu Y. et al., 2024). Additionally, KCs express high levels of pattern recognition receptors, such as mannose receptors. NPs modified with ligands targeting these receptors can achieve specific localization to KCs, enhancing their therapeutic efficacy (Ergen et al., 2017).

HSCs primarily function in lipid and retinol storage. In healthy livers, HSCs remain quiescent. However, under pathological conditions, various inflammatory insults can activate HSCs, leading to their differentiation into fibroblasts (Taru et al., 2024). Prolonged chronic injury results in the activation and proliferation of HSCs, which fill the Disse space, causing hepatic fibrosis (Horn and Tacke, 2024; Che et al., 2023). This pathological process significantly impedes the entry of NPs into the liver parenchyma. Therefore, mitigating HSCs activation to alleviate fibrosis represents an effective therapeutic strategy. Studies have utilized HSCs membrane-derived biofilms to encapsulate drugs into uniform NPs. By leveraging the principle of homologous targeting, these NPs deliver drugs specifically to HSCs, thereby addressing hepatic fibrosis (Cheng et al., 2024). Another approach involves modifying NPs with vitamin A, capitalizing on the characteristic ability of HSCs to store vitamin A to target drug delivery directly to these cells (Niu et al., 2023).

LSECs, uniquely positioned at the interface of blood and hepatocytes, play a pivotal role in regulating the passage of substances into the liver parenchyma (Mcconnell et al., 2023). Central to understanding NP-LSEC interactions is the concept of liver fenestrations. The diameter of these fenestrations imposes physical constraints on the entry of NPs into the liver, necessitating meticulous consideration of NP size during the design phase (Gage et al., 2020). By tailoring NP dimensions to align with the specific fenestration sizes of target species or patient populations, researchers can optimize liver targeting efficiency, thereby enhancing therapeutic outcomes. Beyond their role in filtration, LSECs serve as crucial antigen-presenting cells, actively participating in immune surveillance within the liver (Hammoutene and Rautou, 2019). Their high expression of mannose receptors underscores their potential as targets for immunomodulatory strategies (Greuter et al., 2022). NPs engineered to specifically bind mannose receptors on LSECs could facilitate targeted delivery of therapeutic agents, thereby enhancing localized treatment effects while minimizing systemic side effects. Recent studies have illuminated the charge-selective properties of LSECs, revealing a preferential uptake of negatively charged NPs compared to their positively charged counterparts (Yap et al., 2020). Capitalizing on this observation, researchers have designed charge-selective NPs that exploit these preferences to achieve heightened liver accumulation. Charge-selective NPs have been shown to effectively suppress endothelial growth factor receptor-2 (EGFR-2) expression in LSECs, a pivotal step in inhibiting angiogenesis and tumor progression in mouse HCC model (Yazdi et al., 2024).

Hepatocytes, being the most abundant cell type in the liver and central to its functional execution, are prime targets for NP-mediated therapeutic interventions (Zhao et al., 2024). One of the prominent strategies for targeting hepatocytes involves exploiting the high concentration of digestive enzymes within these cells. Many studies have demonstrated that targeting specific digestive enzyme receptors on hepatocytes to achieve efficient delivery of therapeutic agents. This approach leverages the natural biological processes of the liver to enhance the specificity and effectiveness of drug delivery systems (Zhao et al., 2024; Zhang et al., 2024c; Lu et al., 2024). Among the various targeting ligands, N-acetylgalactosamine (GalNAc) and apolipoprotein E (ApoE) stand out for their exceptional affinity and functionality in hepatocyte targeting. GalNAc binds with high specificity to the ASGPR expressed on the surface of hepatocytes (He X. et al., 2024). This interaction facilitates the internalization of NPs, making it an attractive strategy for delivering drugs or genes directly to hepatocytes. By conjugating GalNAc to the surface of NPs or therapeutic cargos, researchers can significantly enhance the uptake efficiency and specificity towards hepatocytes, thereby improving therapeutic outcomes (Kim et al., 2022). Similarly, ApoE, a major apolipoprotein found in chylomicrons, plays a crucial role in lipid metabolism and is readily taken up by hepatocytes. Anchoring ApoE onto NP surfaces has been shown to enhance the targeting efficiency of NPs to hepatocytes, offering another viable strategy for liver-specific drug delivery (Lyu et al., 2024). This approach not only improves the bioavailability of drugs at the target site but also minimizes systemic side effects by reducing off-target accumulation (Kim et al., 2021).

## 3 Factors affecting the biodistribution of NPs

**FIGURE 3:**
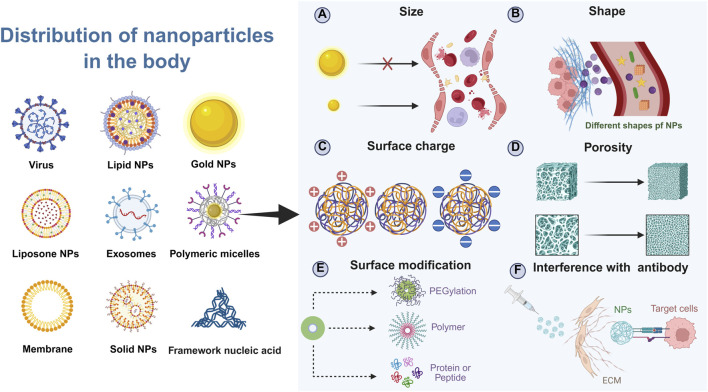
Schematic illustration of factors affecting the biodistribution of NPs in the body. Created in https://BioRender.com.

The biodistribution of NPs after intravenous, intramuscular, or intraperitoneal injection is a critical determinant of their therapeutic efficacy and safety. Several key physicochemical properties of NPs significantly impact their ability to be taken up by the liver (Figure 3). The size of NPs is a primary determinant of their fate within the body (Zaleski et al., 2024). Larger NPs often face difficulties in crossing the fenestrations of LSECs, whereas smaller NPs can more readily pass through these openings. Additionally, the shape of NPs plays a crucial role, spherical NPs typically exhibit different uptake kinetics compared to rod-shaped or other anisotropic forms (Yan et al., 2024). Furthermore, the surface charge of NPs also affects their interaction with cellular membranes and their subsequent internalization by hepatocytes (Dykman et al., 2025). The surface modification of NPs is another critical factor influencing their hepatic uptake. Functional groups or ligands attached to the NP surface can enhance or inhibit interactions with specific cell types within the liver (Dykman et al., 2025). The surface chemistry of NPs, modified with specific functional groups or targeting ligands, profoundly influences their interactions with biological systems. For instance, the incorporation of polyethylene glycol is a common strategy to impart “stealth” properties and prolong circulation time by reducing opsonization and mononuclear phagocyte system uptake (Liu et al., 2024). The material composition of NPs, including their hardness and porosity, can also influence their absorption and distribution within the liver. Stiffer materials may resist deformation during passage through the narrow fenestrations of while porous NPs could allow for better interaction with cellular components (Kent et al., 2024). Understanding the factors that influence how NPs are taken up and utilized by the liver is essential for optimizing their design and application in liver-targeted therapies.

### 3.1 Size

The size of NPs is a critical determinant of their administration routes and biodistribution within the body. The clearance mechanisms in the bloodstream play a significant role in determining the fate of NPs based on their size. Larger NPs, typically those exceeding 500 nm in diameter, are prone to rapid clearance by macrophages in the blood. This clearance process limits their circulation time and availability for targeted delivery, making them less suitable for systemic applications (Schofield et al., 2024). In contrast, micrometer-sized NPs (>1 μm) are often utilized in pulmonary drug delivery systems, particularly through nebulization (Witten et al., 2024; Li X. et al., 2024). These larger particles are generally not recommended for intravenous administration due to their inability to navigate the intricate network of capillaries and the risk of embolism. At the other end of the spectrum, extremely small NPs (<50 nm) exhibit enhanced tissue penetration capabilities. Their small size allows them to distribute throughout the body more effectively but also increases the likelihood of uptake by lymphatic tissues (Rong et al., 2024; Feng T. et al., 2024). Additionally, these tiny NPs can be filtered and eliminated by the kidneys. The glomerular endothelial cells in the kidneys have fenestrations smaller than 10 nm, which means that NPs below this size threshold will be rapidly cleared from the bloodstream via renal excretion (He Y. et al., 2024; Li et al., 2025). Consequently, ultra-small NPs have very short circulation times, often being completely excreted within a few hours after administration.

Given these considerations, the ideal size range for NPs intended for liver targeting falls between 50 nm and 200 nm. They are large enough to avoid immediate renal excretion, thereby prolonging their circulation in the bloodstream (Moghimi et al., 2023). This extended half-life is crucial for allowing sufficient time for the NPs to extravasate and accumulate within the liver (Bussin et al., 2025). More specifically, the fenestrated endothelial lining of the liver sinusoids contains pores ranging from 50 to 150 nm in diameter. NPs under 200 nm can efficiently traverse these fenestrations, gaining direct access to the space of Disse and subsequently to the underlying hepatocytes. This process is fundamental for passive targeting and enhanced permeability.

Furthermore, this size range is highly compatible with various active targeting strategies. For instance, NPs can be functionalized with ligands such as galactose to target the asialoglycoprotein receptor (ASGPR) abundantly expressed on hepatocytes (Feng et al., 2025). The 50–200 nm size ensures that these ligand-decorated particles maintain favorable pharmacokinetics and biodistribution, maximizing receptor-mediated internalization while minimizing non-specific off-target accumulation (Bai et al., 2025). In summary, the 50–200 nm size range strikes a critical balance between prolonged circulation, efficient hepatic penetration, and enhanced cellular uptake, making it a cornerstone rationale in the design of liver-targeted nanotherapeutics.

### 3.2 Shape

The shape of NPs significantly influences their biodistribution and cellular uptake, particularly within the liver. Among various shapes such as cubes, rods, spheres, and stars, spherical NPs are particularly advantageous for cellular uptake, especially under conditions of slow blood flow typical in hepatic sinusoids (Ko et al., 2024). In turbulent blood flow, spherical NPs experience distinct forces compared to other shapes. They tend to be compressed by the walls of smaller radius vessels and subsequently move towards larger radius vessels. This movement facilitates their eventual internalization by endothelial or other cell types lining these vessels (Hu G. et al., 2024). In contrast, non-spherical NPs, such as nanorods or cubic structures, exhibit markedly different behaviors in turbulent flow. Their anisotropic shapes lead to complex rotational dynamics and increased susceptibility to collisions with vascular walls. Rather than undergoing efficient cellular uptake, these particles often enter a sliding motion along the vessel centerline, reducing their contact time with the endothelial surface and diminishing their likelihood of being engulfed via endocytic pathways (Bottger et al., 2020; Lozano-S et al., 2024). This hydrodynamic profiling underscores the importance of shape uniformity in minimizing off-target movement and maximizing hepatic accumulation.

Furthermore, the preference for spherical NPs is reinforced by biological interactions at the cellular level. Their symmetrical shape allows for more uniform ligand distribution, which is crucial for receptor-mediated uptake mechanisms prevalent in liver cells (Kent et al., 2024; Bartneck, 2021). The combination of favorable hemodynamic properties and optimized surface presentation makes spherical NPs particularly advantageous for navigating the liver’s intricate vasculature and achieving efficient intracellular delivery (Teng et al., 2023; Yue et al., 2023). Thus, while novel shapes may offer unique mechanical or optical properties, spherical nanoparticles remain the gold standard for hepatic targeting due to their enhanced hydrodynamic performance and cellular engagement in slow-flow systems (Arjunan et al., 2024).

This hydrodynamic effect is exacerbated in diseased states such as hypertension and atherosclerosis, where increased turbulence and narrowing of blood vessels are common (Tao and Salmeron, 2024; Costantini et al., 2024). The altered blood flow patterns in these conditions can further hinder the effective delivery and uptake of non-spherical NPs, highlighting the importance of considering NP shape in the design of targeted therapies.

### 3.3 Charge

The surface charge of NPs is another critical factor influencing their biodistribution and tissue-specific accumulation. Here, we explore how the presence of surface charges affects the apparent acid dissociation constant (pKa) of NPs, which in turn influences their aggregation properties in different tissues. Research has demonstrated that the liver preferentially accumulates NPs with a pKa between 6 and 7. Conversely, lungs exhibit a higher affinity for NPs with a pKa greater than 9. NPs with a pKa less than 6 are more readily taken up by the spleen. These findings suggest that the pKa of NPs can be strategically manipulated to enhance their uptake by specific organs (Pilkington et al., 2021; Cheng et al., 2020).

One sophisticated strategy to engineer NPs for improved hepatic accumulation involves the deliberate modulation of their surface charge through the conjugation of specific biological proteins. A prominent example is the use of ApoE, a protein that naturally carries a net negative charge and plays a key role in lipid metabolism and receptor-mediated endocytosis. When ApoE is adsorbed or covalently attached to the surface of synthetic NPs that are typically engineered to possess an initial positive charge, it fundamentally alters their electrostatic profile. This conjugation effectively neutralizes the highly positive surface and confers a negatively charged, biomimetic corona. This newly acquired negative surface characteristic is critically important for targeting LSECs. These resident liver cells exhibit a well-documented affinity for and efficiently scavenge negatively charged macromolecules and particulates from the circulation, a process driven by specialized scavenger receptors.

Beyond merely facilitating initial LSEC recognition and acceptance, the ApoE corona acts as a sophisticated biological targeting ligand. It enables the NPs to hijack endogenous metabolic pathways, particularly those involving the LDL receptor family abundantly expressed on the surface of hepatocytes. Consequently, ApoE-functionalized NPs benefit from a dual-targeting mechanism: initial sequestration by LSECs due to charge preference, followed by enhanced, receptor-mediated uptake into hepatocytes. Empirical evidence strongly supports the efficacy of this approach. For instance, comprehensive studies utilizing gold nanoparticles with a diameter of approximately 80 nm—a size optimized for traversing hepatic sinusoidal fenestrations—demonstrate a dramatic increase in liver accumulation when coated with ApoE. Quantitative biodistribution analyses reveal that these bio-functionalized NPs achieve significantly higher concentrations within liver tissue compared to their uncoated, positively charged counterparts, which are more prone to opsonization and clearance by the immune system or accumulation in off-target organs (Schottler et al., 2016; Saha et al., 2016). Thus, ApoE modification represents a powerful protein-based strategy to leverage the liver’s inherent cellular and molecular machinery for superior nanoparticle delivery.

One approach to designing NPs for enhanced liver uptake involves modifying their surface charge through conjugation with proteins. For instance, ApoE, a protein commonly associated with negative charges, can be used to modify positively charged NPs. This modification alters the overall charge characteristics of the NPs, making them more attractive to LSECs, which have a preference for negatively charged particles. Furthermore, ApoE not only facilitates the acceptance of NPs by LSECs but also enhances their uptake by hepatocytes. Studies have shown that gold NPs coated with ApoE and measuring 80 nm in diameter achieve significantly higher concentrations within the liver compared to uncoated NPs.

### 3.4 Surface modification

Surface modification of NPs is a common strategy for liver targeting, serving two primary purposes: enhancing uptake by specific cells within the liver and evading rapid clearance by the body (Yang M. et al., 2024). One effective approach to prevent rapid uptake and clearance of NPs is surface modification with polyethylene glycol (PEG). PEGylation significantly increases the hydrophobicity of NPs, preventing their recognition and subsequent clearance by blood macrophages. This modification extends the half-life of NPs in the bloodstream, thereby enhancing their circulation time and potential for targeted delivery (Li H. et al., 2024; Zheng et al., 2023). Another method to avoid rapid NP clearance involves coating NPs with a biomimetic membrane. Coating nanoparticles with biomimetic membranes—such as those derived from red blood cells, platelets, or leukocytes—has emerged as a powerful strategy to enhance biocompatibility and impart advanced targeting capabilities. The use of such membranes can mimic endogenous substances, signaling to cells that the NPs are non-threatening and thus less likely to be cleared by immune responses. This biomembrane coating provides a form of immunological camouflage, facilitating longer systemic circulation of NPs (Mizuta et al., 2024; Lin et al., 2024). Conjugating antibodies to the NP surface is another effective strategy for promoting targeted delivery. For instance, synthesizing an antibody targeting epidermal growth factor receptor-2 (HER2) and anchoring it to NPs allows for specific targeting of HER2-positive tumor cells. This targeted approach enhances the selective accumulation of NPs at the desired site, improving therapeutic efficacy while minimizing off-target effects (Guo et al., 2024; Ma et al., 2022).

## 4 Applications of NPs in liver diseases

**FIGURE 4:**
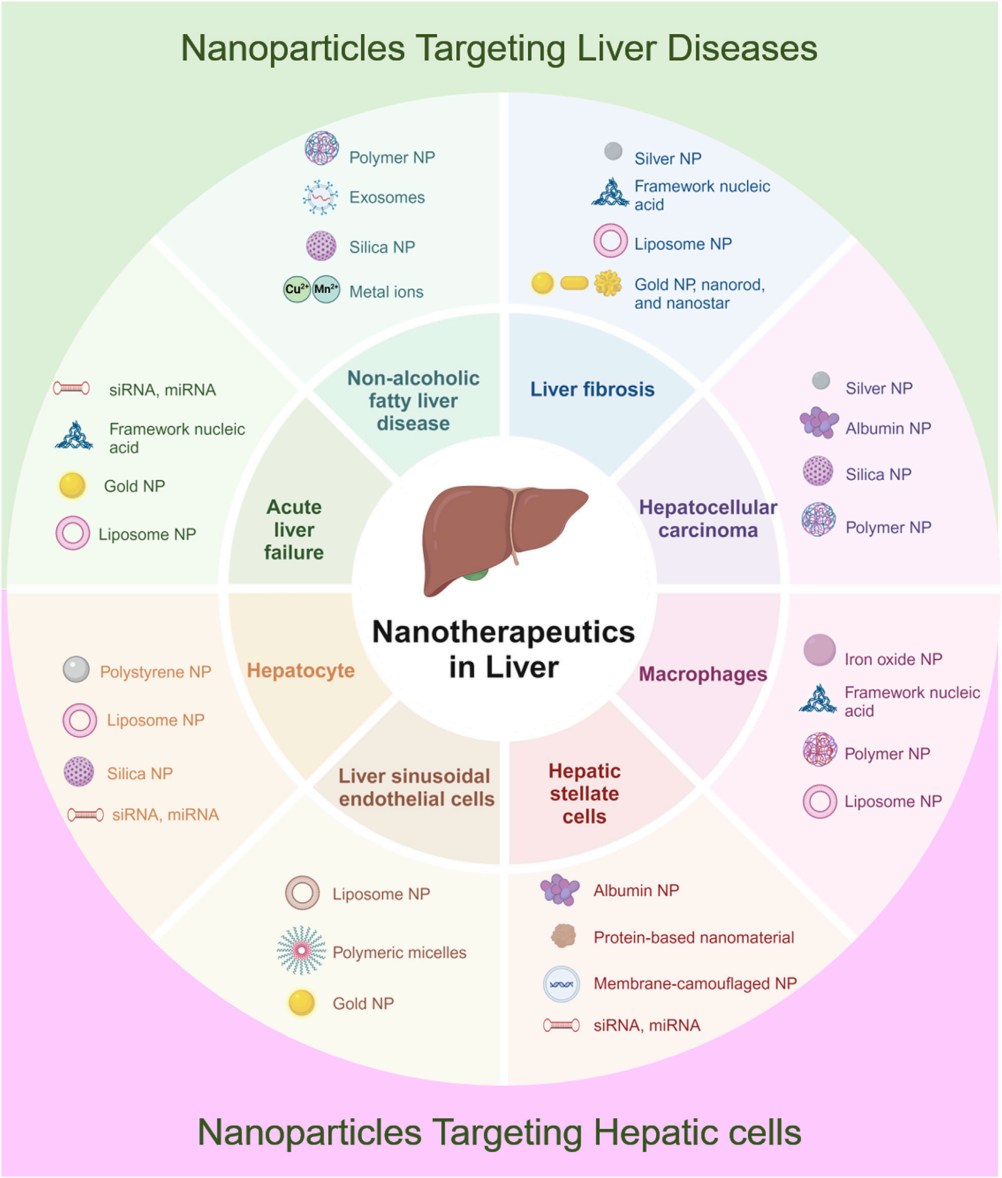
Schematic illustration of applications of NPs in diverse liver diseases and hepatic cells. Created in https://BioRender.com.

The accumulation characteristics of NPs within the liver and their interactions with various hepatic cells have been extensively studied, highlighting the potential applications of NPs in liver diseases. Given that each liver disease has a distinct pathogenesis, the design and targeting strategies for NPs vary significantly across different liver disorders. This section will summarize the pathological mechanisms of common liver diseases and discuss the applications of NPs in these conditions (Table 2). Figure 4 illustrates the diverse applications of NPs across various liver diseases and liver cell types.

**TABLE 2:** Applications of NPs to treat different liver diseases.

Diseases	Targeted cells	Targeting approaches	Vector	Administration route	Animal model	Efficiency	References
ALF	Macrophages	Scavenger receptor-A	Palmitic acid-modified serum albumin	Intravenous	Mice	More than 50% NPs are taken up in liver, and the control group has almost no NPs uptake	Zhang et al. (2023a)
ALF	Hepatocyte	Antioxidant nanozyme-hepatocyte-like cells	N-acetylcysteine-capped gold nanoclusters, forming the N–Au@hydrogel	Intraperitoneal	Mice	The HS/N–Au@composite group also demonstrated the most favorable reductions in AST and ALT serum levels, effectively suppressing by 12.32-fold and 10.20-fold, respectively, compared to the control model group	Jin et al. (2025)
ALF	Hepatocyte	Conjugating acid-cleavable hydrophobic moieties to maltodextrin	Ketalized maltodextrin	Intravenous	Mice	A majority (∼70%) of drug payloads was released at 24 h	Go et al. (2018)
ALF	Macrophages	Red blood cell membrane	Mesenchymal stem cells inspired biomimetic nanoframework	Intravenous	Mice	The final biomimetic nanostructure had a loading capacity of 6.98% for rhein and 7.51% for freezedried MSC-conditioned medium	Feng et al. (2024b)
ALF			Porous silicon, gold NPs	Intravenous	Mice	More than 50% DPSi/DAu@AcDEX are taken up in liver, and the control group has almost no NPs uptake	Liu et al. (2018)
ALF	Hepatocyte	Bovine serum albumin	Bilirubin and 18β-Glycyrrhetinic acid	Intravenous	Mice	B/BG@N expressed abundant luciferase activity in hepatocytes, while the control group has almost no luciferase uptake	Yao et al. (2023)
ALF	Macrophages	Macrophage membrane	PLGA NPs	Intravenous	Mice	MVs-DiD-NPs were observed with the highest fluorescence intensity in liver tissue	Shang et al. (2023)
ALF	Macrophages	Manganese porphyrin via π-π stacking interaction with G-quadruplex	DNA nanoplatform	Intravenous	Mice	TDN-siTNF-α/-G4-MnP4 shows almost complete liver accumulation after within 2 h	Wei et al. (2024a)
ALF	Macrophages	Phenylboronic acids	PEGylated, phenylboronic-acid-protected L-DOPA precursor NPs	Intravenous	Mice	PADN treatment displayed obvious efficacy in reducing AST levels over control group	Zhao et al. (2021)
NAFLD	Hepatocyte	Rubicon‐specific CRISPR‐Cas9 components	Lipid NPs	Intravenous	Mice	≈90% of the NPs accumulated in the liver, while only ≈5% were detected in the spleen	Bai et al. (2024a)
NAFLD	Hepatic stellate cells	CD44-targeting glycosaminoglycan biopolymer	Hyaluronic acid-bilirubin NPs	Intravenous	Mice	Higher fluorescence signals from HABN–Cy5.5 were found in the liver of mice kept on a CD-HFD than in their other organs	Shinn et al. (2024)
NAFLD	Hepatocyte	Albumin	Natural compound ginsenoside compound K	Intravenous	Mice	NabCK supplementation increased fecal cholesterol and cholestanone by 2.86 and 56.32 times, respectively	Yue et al. (2023)
NAFLD	Hepatic stellate cells	Vitamin A	Aminoethyl anisamide coated in NPs	Intravenous	Mice	siRNA@Cy5.5NP-AEAA5% exhibited greater accumulation in fibrotic livers compared with nontargeted siRNA@Cy5.5NP	Zhang et al. (2023b)
NAFLD			TiO2, Au, and NaYF4 NPs	Oral administrations	Mice	Relative Ces2h mRNA expression of db/db mice increased by ∼2.9–3.3 times, depending on the types of NPs	Cai et al. (2023)
NAFLD	Hepatocyte	Fluorineted polyesters	Biodegradable acid-activated acidifying NPs	Intravenous	Mice	Rhodamine-labeled PEFSU acNPs are rapidly taken up in HepG2 cells with 80% of the HepG2 cells possessing Rho-acNPs within 4 h	Zeng et al. (2023)
NAFLD	Macrophages	Prohibitin binding peptide	Hemin‐ or CoPP‐loaded poly NPs	Intravenous	Mice	PBP‐NPs were highly distributed in the fatty liver of the NASH model than what was observed in the liver of the T2DM model	Hong and Kim (2022)
Liver fibrosis	Hepatic stellate cells	IL-11 scFv	Lipid NPs	Intravenous	Mice	^Cy5^AA3G LNP exhibited a higher and more specific distribution in the liver compared to the nontargeted ^Cy5^AA3 LNP.	Zhang et al. (2024d)
Liver fibrosis	Hepatic stellate cells	Platelet membranes and hepatic stellate cell membranes	Poly (lactic-co-glycolic acid) @Melatonin	Intravenous	Mice	The fluorescence intensity observed with HSCM@PLGA@Cy7.5 was significantly higher than that of PLGA@Cy7.5, at all-time points	Bai et al. (2024b)
Liver fibrosis	Liver sinusoidal endothelial cells and hepatic stellate cells	Chondroitin sulfate	Vismodegib-loaded NPs	Intravenous	Mice	Targeting ability of chondroitin sulfate and vismodegib to the highly expressed receptors in liver	Zhang et al. (2024e)
Liver fibrosis	Hepatic stellate cells	Ligands targeting M6P/IGF-II receptors	Dibenzocyclooctyne functionalised crosslinked micelles	Intravenous	Mice	The mannose-conjugated micelles and retinol-conjugated micelles exhibited consistent trends between *in vitro* and *in vivo* experiments	Balaji et al. (2023)
Liver fibrosis	Macrophages	Ligands targeting RNF41	Dendrimer-graphite NPs	Intravenous	Mice	An intense fluorescence signal in CD11b+ macrophages corresponding to the plasmid EGFP reporter	Moreno-Lanceta et al. (2023)
Liver fibrosis			Self-assembling antagonist peptides NPs	Intravenous	Mice	The fluorescence of the DiR-F-NPs was mainly concentrated on the liver sites	Li et al. (2023a)
Liver fibrosis	Hepatic stellate cells	CREKA (a specific ligand of fibronectin) and chondroitin sulfate (CS, a major ligand of CD44)	Lipid NPs	Intravenous	Mice	CCR NPs showed the highest fluorescent signal in CCl4-induced liver	Li et al. (2023b)
Liver fibrosis	Hepatic stellate cells	Bilirubinn	PEGylated NPs	Intravenous	Mice	Cy5.5@GBRNP fluorescence in the liver was 1.8-fold greater than that in the free Cy5.5 group and 1.5-fold compared to the Cy5.5@BRNP group	Li et al. (2023c)
HCC		Decoy receptor 3 antibodies	PEGylated paramagnetic NPs	Intravenous	Mice	Following coupling with the DCR3 antibody, the Fe NPs-DCR3 group exhibited more effective enrichment at liver tumor sites compared to the Fe NPs group	Jia et al. (2024)
HCC	Heaptocellular carcinoma cells	FIDAS-5, macrophage membrane, and anti-PD-L1	Hollow mesoporous manganese dioxide (MnO_2_) NPs	Intravenous	Mice	After treatment with MF, MFM, and MFMP, relatively high fluorescence was observed in the liver while pronounced fluorescence was only detected in the tumors of the MFMP groups	Zhu et al. (2024)
HCC	Heaptocellular carcinoma cells	Calcium-based thermosensitizer	CaCO_3_ NPs	Intravenous	Mice	Compared with the mice in the PBS group and the IR780 group alone, DMXAA@CBTNps arriving at the tumor site at 24 h still retained more NPs located in the tumor	Zeng et al. (2025)
HCC	Heaptocellular carcinoma cells	Ultrasound-magnified multienzyme-mimicking properties	Ultrasmall Bi_2_Sn_2_O_7_ nanozyme NPs	Intratumoral and Intravenous injection	Mice	Control group and BSO group maintained normal cell tissue morphology, whereas both intravenous and intratumoral injection of BSO + US group resulted in evident histopathological damage	Wei et al. (2024b)
HCC	Heaptocellular carcinoma cells	Man-DSPE-mPEG_2K_ modified with mannose	DOTAP, DOPE, Cho, DSPE-mPEG_2K_, and HMME form liposomes	Intravenous	Mice	The maximum accumulation level in the MLip_Cy5-siBcl-2_ group was significantly higher than that in the Lip_Cy5-siBcl-2_ group, indicating good *in vivo* targeting of MLip_Cy5-siBcl-2_	Wang et al. (2024c)
HCC	CD8^+^ T cells	PD1 proteins	Calcium phosphate NPs	Intravenous	Mice	siPDL1‐CaP@PD1‐NVs exhibited significantly higher fluorescent intensity in tumor than that of siPDL1‐CaP@NVs without PD1‐expressing on the cell membrane, indicating an efficient tumor targeting ability of siPDL1‐CaP@PD1‐NVs	Sun et al. (2024)
HCC	Heaptocellular carcinoma cells	Cationic poly (l-lysine) complexing anti-MFAP-5 siRNA	Polypept(o)ide-based polyion complex micelles	Intravenous	Mice	At 24 h post-administration, Cy5.5 signal from siCy5.5 PICMs and siCy5.5/DES PICMs was mainly located in the liver region, while the signal of siCy5.5-loaded in LNPs began to fade and was less localized in the liver region	Schneider et al. (2024)

### 4.1 Applications of NPs in acute ALF

ALF is a critical condition characterized by the sudden and severe impairment of liver function in individuals with no pre-existing liver disease. The hallmark features of ALF include coagulopathy and hepatic encephalopathy, with extensive hepatocellular necrosis observed histologically. The pathogenesis of ALF involves extensive hepatocyte death, leading to the rapid decline in liver function. Viral infections, such as those caused by hepatitis B and C viruses, can trigger an immune response that results in hepatic inflammation and necrosis. Drug-induced liver injury occurs when certain medications or toxins directly damage hepatocytes or induce an immune-mediated reaction. Autoimmune hepatitis involves the body’s immune system attacking liver cells, causing chronic inflammation and acute deterioration. Currently, liver transplantation remains the most effective treatment for ALF; however, the scarcity of donor organs presents a significant challenge. Although artificial liver support systems can mitigate the progression of ALF to some extent, the mortality rate remains high.

**FIGURE 5:**
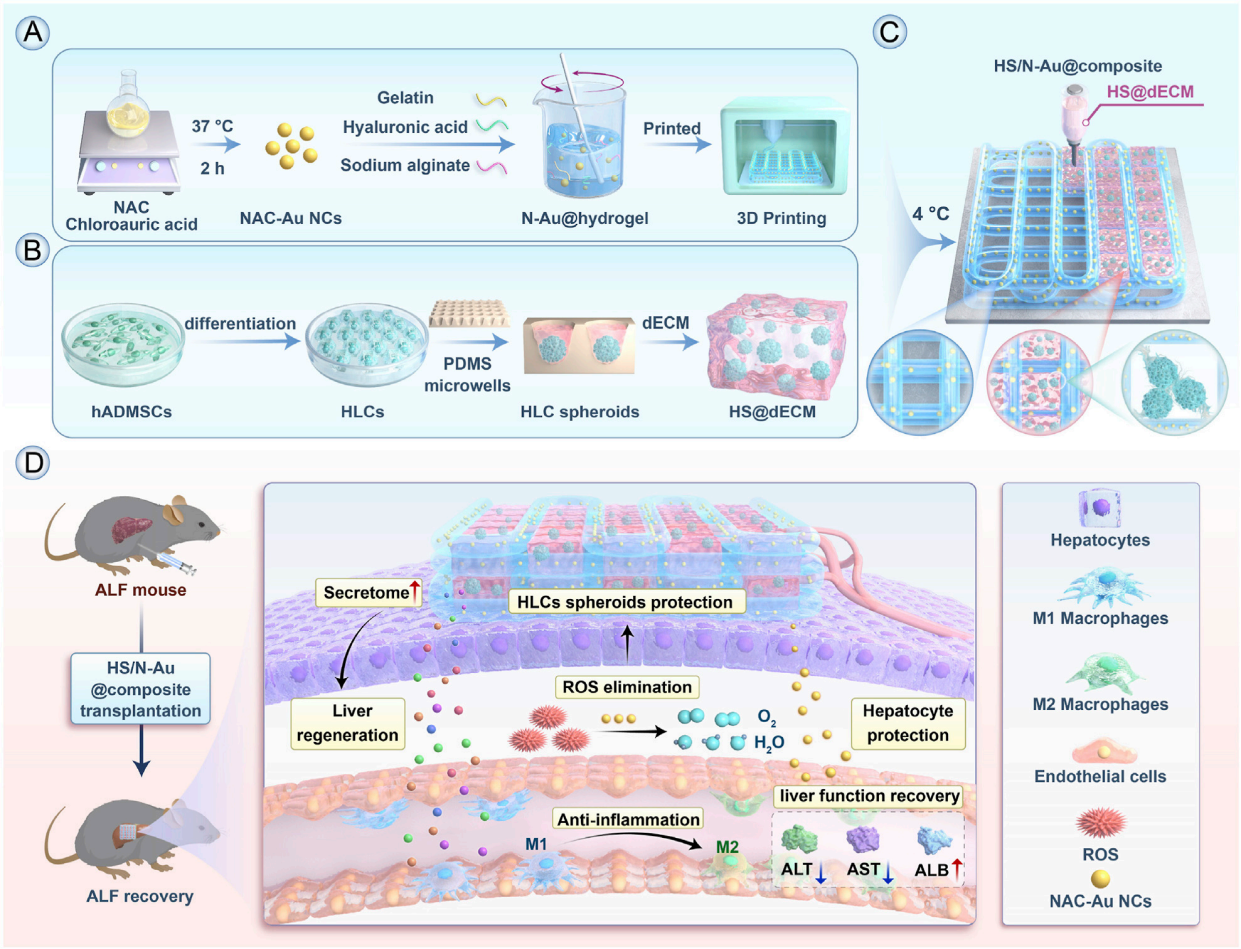
Alleviation of ALF by HS/N–Au@composite through differentiation of macrophages from M1 to M2. **(A)** Preparation of N–Au@hydrogel. **(B)** Construction of decellularized ECM (dECM)-based hydrogel (HS@dECM). **(C)** Assembly of the HS/N–Au@composite. **(D)** *In vivo* treatment of acute liver failure using the composite, showing functional liver recovery (Jin et al., 2025). Copyright from Elsevier.

NPs offer promising platforms for targeted therapy in ALF, especially in controlling the expressive inflammation in macrophages and promoting the regeneration of hepatocytes. For ALF, rapid intervention is critical, and nucleic acid-based nanoparticles, gold nanoparticles, and Lipid Nanoparticles have been most prevalent due to their excellent efficacy in gene silencing, anti-oxidative stress, and rapid hepatocyte uptake (Figure 4). One notable study involved the synthesis of SchB-PSA NPs, which were created by modifying palmitic acid-modified serum albumin with scavenger receptor-A (SR-A). These SchB-PSA NPs exhibited significant therapeutic potential by inhibiting the NF-κB pathway in macrophages and reducing hepatocyte necrosis, thereby lowering mortality rates in ALF mouse models (Zhang R. et al., 2023). Another innovative approach involves the use of gold NPs (AuNPs) within a 3D-printed hydrogel scaffold, encapsulating NAC-modified AuNPs to form HS/N–Au@composite. This composite targets necrotic areas in the liver, clears reactive oxygen species (ROS) within macrophages, and promotes the differentiation of macrophages from the M1 subtype to the M2 subtype, offering a novel strategy for ALF treatment (Figure 5) (Jin et al., 2025). Furthermore, ketalized maltodextrin (KMD) NPs leverage pH-sensitive properties to release therapeutic drugs in the acidic regions of necrotic hepatocytes. This targeted delivery mechanism enhances ultrasound imaging capabilities and provides an effective means for targeting ALF treatment (Go et al., 2018). These studies collectively highlight the versatility and efficacy of NPs in addressing the complex pathologies associated with ALF.

### 4.2 Applications of NPs in NAFLD

As lifestyle changes contribute to the global rise of obesity and type 2 diabetes, the prevalence of NAFLD is significantly increasing. NAFLD often presents with subtle symptoms but can progress histologically to non-alcoholic steatohepatitis (NASH), which may further develop into advanced liver disease, cirrhosis, and hepatocellular carcinoma (Younossi, 2019). Statistics indicate that the global incidence of NAFLD is approximately 24% (Huang et al., 2021). Predictably, as viral hepatitis is effectively controlled, NAFLD will impose a substantial health burden and economic cost (Powell et al., 2021). Lifestyle modification remains the most effective and fundamental approach to treating NAFLD, yet it is challenging to implement (Demir et al., 2022). Given the rapid global increase in NAFLD and NASH, developing early treatment strategies for these conditions is of paramount importance.

**FIGURE 6:**
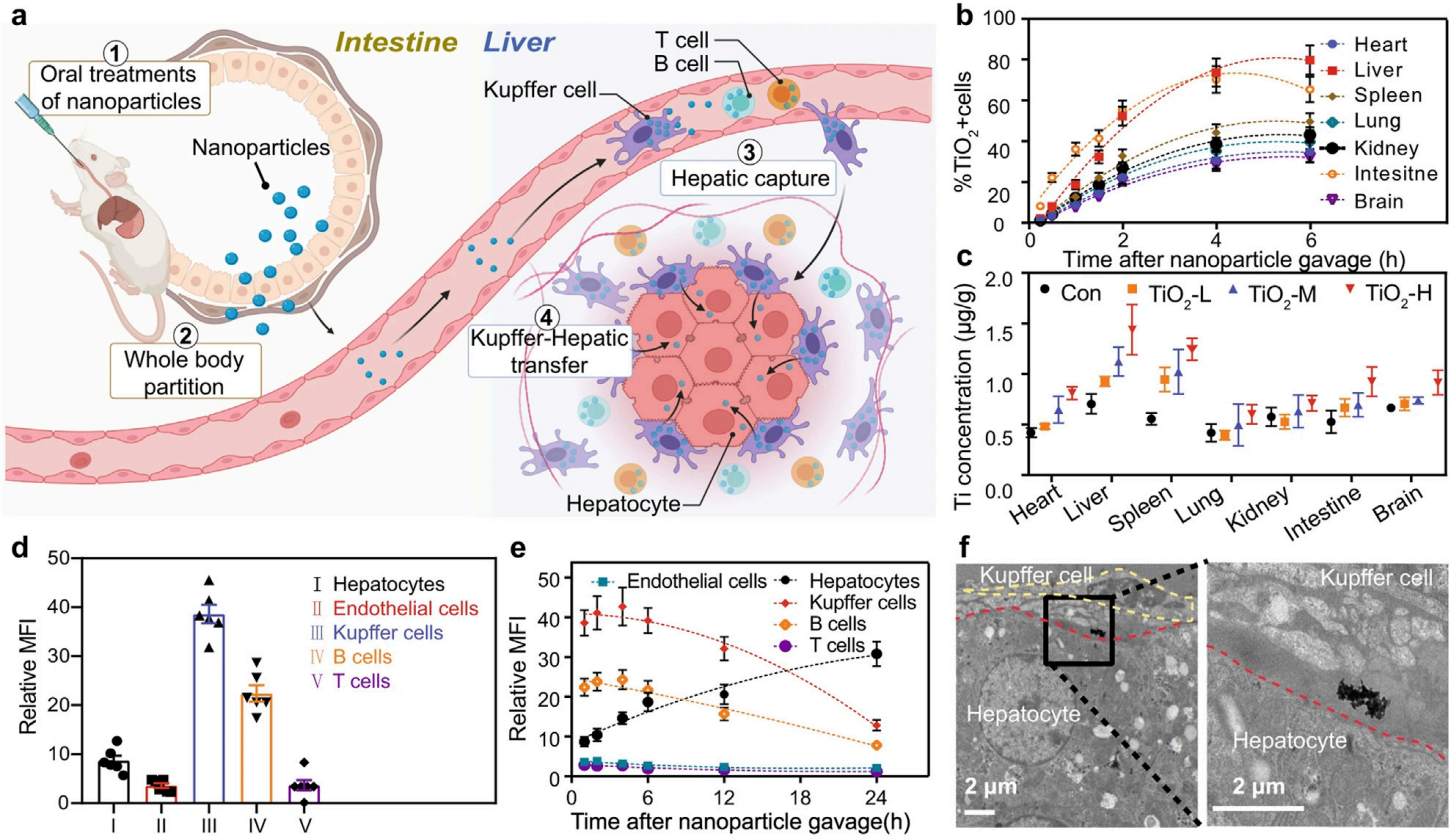
Administration of TiO2, Au, and NaYF4 to treat NAFLD, **(a)** Schematic of oral NP administration and liver targeting. **(b)** Time-dependent NP cell distribution in major organs. **(c)** Ti element biodistribution varying doses. **(d,e)** NP accumulation in liver cell types by MFI. **(f)** TEM images showing Kupffer cell-to-hepatocyte NP transfer (Cai et al., 2023). Copyright from Nature.

Macrophages play a crucial role in the development of NAFLD, making them a primary target for NPs-based therapies (Govaere et al., 2022). In the context of NAFLD, which often requires long-term management, polymeric NPs, silica nanoparticles, and exosomes have shown great promise for their sustained drug release profiles, high biocompatibility, and ability to modulate chronic inflammatory pathways (Figure 4). Given the chronic nature and long-term treatment requirements of NAFLD, oral administration is considered the most effective route for delivering NPs (Kulchar et al., 2023). Studies comparing the hepatic uptake efficiency of orally administered TiO2, Au, and NaYF4 have demonstrated that these common NPs can effectively enter the liver. Further pathological analyses suggest that these NPs can reduce lipid accumulation and steatosis in hepatocytes, indicating promising applications for NPs in NAFLD treatment (Figure 6) (Cai et al., 2023). Previous research has implicated Rubicon in the progression of NAFLD, suggesting that targeting this pathway could be beneficial (Tanaka et al., 2016). Consequently, encapsulating CRISPR-Cas9 components targeting Rubicon within NPs has emerged as a potential therapeutic strategy. Results indicate that a single injection of NPs targeting Rubicon can enhance lipid metabolism in hepatocytes and alleviate NAFLD (Bai et al., 2024a). Traditional Chinese medicine has shown significant advantages in treating chronic diseases. Researchers have developed sustained-release NPs composed of ginsenoside compound K (CK) and albumin, known as nabCK. Animal models suggest that nabCK can promote lipid homeostasis in hepatocytes by reducing mTOR activation (Yue et al., 2023).

### 4.3 Applications of NPs in liver fibrosis

Liver cirrhosis is a stage in the progression of liver fibrosis. Clinically, cirrhosis is classified into compensated and decompensated phases. Once patients enter the decompensated phase, they exhibit symptoms of liver failure and portal hypertension, significantly impacting their quality of life (Caligiuri et al., 2021). Over the past few decades, viral hepatitis-induced cirrhosis has been somewhat controlled etiologically. However, once cirrhosis develops, it remains an irreversible progression towards liver failure or HCC (Lai and Afdhal, 2019). Mechanistically, cirrhosis primarily results from inflammation promoting the activation of hepatic stellate cells, driving the deposition of extracellular matrix into fibrotic tissue. Therefore, inhibiting macrophage inflammation, reducing the recruitment of bone marrow mononuclear cells, or controlling the activation of stellate cells could potentially mitigate liver fibrosis (Kisseleva and Brenner, 2021).

**FIGURE 7:**
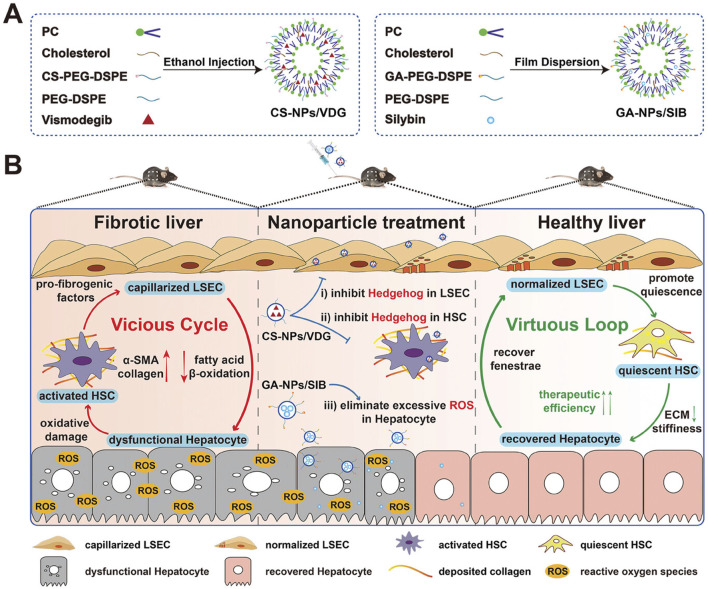
Mechanism of the vicious cycle-breaking system promoting liver fibrosis reversal. **(A)** Preparation of CS-NPs/VDG and GA-NPs/SIB; **(B)** The treatment initiates a virtuous loop: normalized LSECs inactivate HSCs via NO signaling; quiescent HSCs degrade ECM; repaired hepatocytes secrete VEGF to maintain LSEC fenestration, collectively restoring liver homeostasis. The application of CS-NPs/VDG and GA-NPs/SIB are able to break the vicious cycle and maintain the function of hepatocyte (Zhang et al., 2024e). Copyright from Wiley.

For Liver Fibrosis, silver nanoparticles for their anti-inflammatory properties, nucleic acid-based NPs for gene therapy, and Lipid Nanoparticles have been the most widely investigated (Figure 4). Fibrotic livers exhibit elevated expression of IL-11 (Zhang C. et al., 2023). Studies have developed antibodies targeting IL-11 and encapsulated their mRNA within AA3G NPs (mIL11-scFv@AA3G). *In vivo* imaging in mice demonstrated the high accumulation of mIL11-scFv@AA3G in the liver. Pathological results indicated a significant reduction in fibrosis levels in mice treated with mIL11-scFv@AA3G (Zhang C. et al., 2024). Oxidative stress damage to hepatocytes is also a crucial factor in fibrosis progression (Gallego-Duran et al., 2024). Researchers loaded melatonin into poly (lactic-co-glycolic acid) (PLGA) and coated it with platelet membranes (PM) and activated hepatic stellate cell membranes (HSCM), creating PM@PLGA@Melatonin and HSCM@PLGA@Melatonin, respectively. Experimental results showed that PM@PLGA@Melatonin and HSCM@PLGA@Melatonin could alleviate oxidative stress and endoplasmic reticulum stress in hepatic stellate cells, thereby reducing fibrosis levels (Bai et al., 2024b). Another study aimed to break the vicious cycle of liver fibrosis formation. The authors constructed chondroitin sulfate-modified and vismodegib-loaded NPs (CS NPs/VDG) to restore HSC homeostasis. Additionally, they prepared glycyrrhetinic acid-modified and silybin-loaded NPs (GA NPs/SIB) to mitigate oxidative stress in hepatocytes. The combined application of these two types of NPs was successfully practiced in a mouse model of liver fibrosis (Figure 7) (Zhang et al., 2024e).

### 4.4 Applications of NPs in HCC

Hepatic tumors represent the final stage of various liver diseases, with HCC being the most common primary liver malignancy (Vogel et al., 2022). HCC exhibits significant heterogeneity at both cellular and molecular levels. Currently, surgical resection and radiofrequency ablation are viable treatment options for early-stage HCC. For advanced HCC, chemotherapy and immunotherapy are employed (Llovet et al., 2022). However, due to the immune-privileged nature of the liver, many chemotherapeutic agents fail to reach the tumor site effectively, severely impacting treatment efficacy and increasing side effects (Llovet et al., 2022; Llovet et al., 2021). This scenario presents a unique opportunity for targeted NPs therapies. NPs offer promising tools for overcoming the challenges associated with liver immunoprivilege and enhancing the delivery of therapeutic agents to the tumor site (Chen L. et al., 2024). By leveraging their unique properties for targeted delivery and imaging, NPs can provide promising treatment strategies.

**FIGURE 8:**
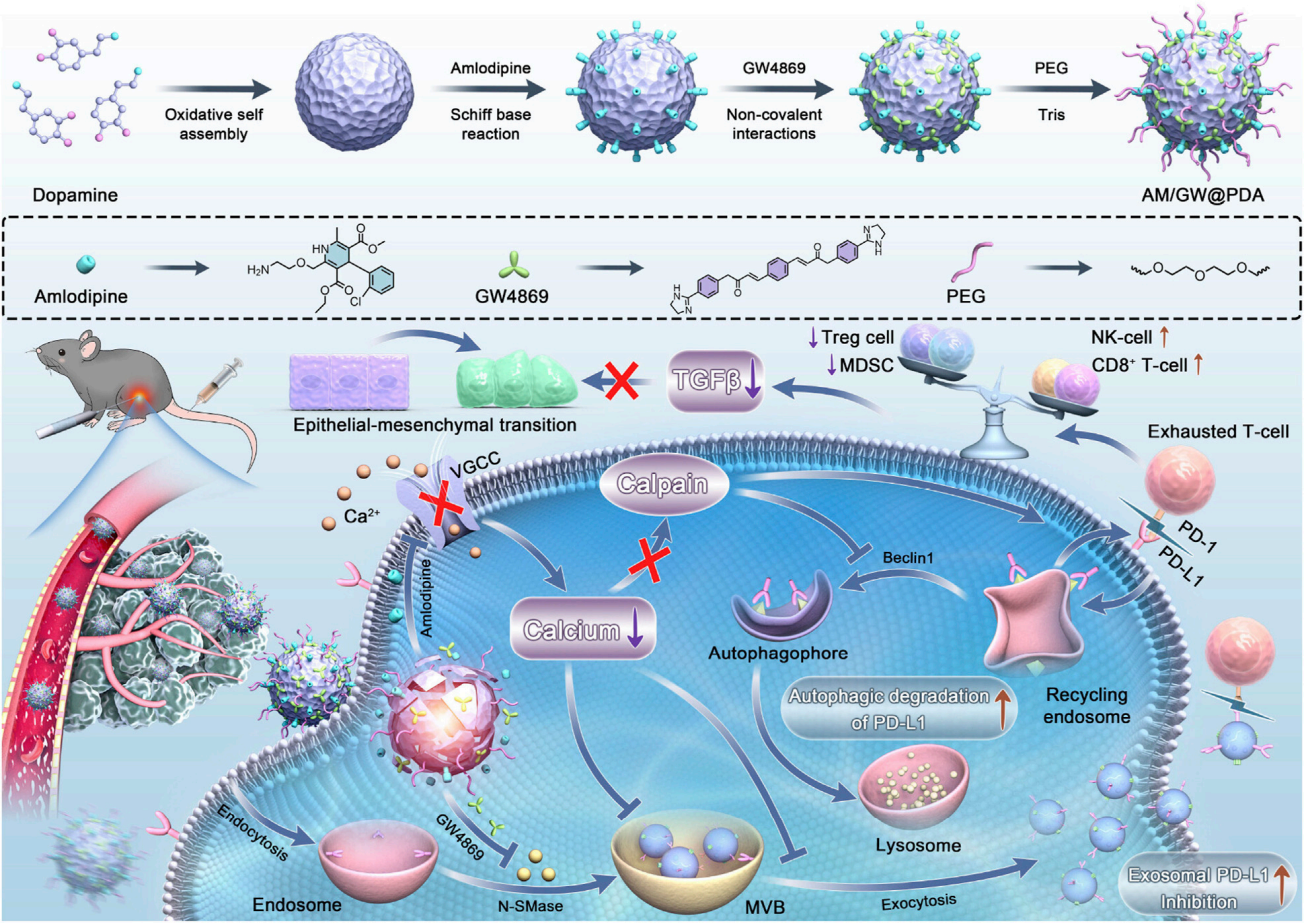
Remodeling the immunosuppressive TME post-iRFA via a polydopamine-based nanomodulator. The platform delivers GW4869 and amlodipine (AM) to suppress exosome biogenesis/secretion and degrade PD-L1. This strategy rejuvenates cytotoxic T cells and NK cells, reduces immunosuppressive cells, and inhibits HCC progression and metastasis (Zhu et al., 2024). Copyright from American Chemical Society.

For HCC, the need for both therapy and imaging has made multifunctional platforms like silver nanoparticles (theranostics), silica nanoparticles (drug delivery), and polymeric NPs (versatile functionalization) the most extensively applied strategies (Figure 4). Recent advances have led to the development of a dual-responsive, magnetism-controlled drug delivery system based on PEGylated paramagnetic NPs coupled with decoy receptor 3 (DCR3). Upon entry into the body, these NPs move along DCR3 gradients to specifically target sites of HCC. They anchor at regions with the highest concentration of DCR3 and inhibit tumor progression (Jia et al., 2024). Given that the function of immune cells such as CD8^+^ T cells is often suppressed in the tumor microenvironment (Llovet et al., 2022), leading to tumor proliferation and metastasis, another study combined GW4869 and amlodipine (AM) using polydopamine nanomodulators to synthesize NPs. This synergistic effect enhances the functionality of intratumoral CD8^+^ T cells and natural killer cells, effectively inhibiting tumor growth (Figure 8) (Zhu et al., 2024). Additionally, leveraging the local temperature differences within tumors (Chen Y. et al., 2024), researchers employed a calcium-based thermal-sensitive enhancer (CBT) for targeted therapy. The chemotherapeutic agent DMXAA was encapsulated within a CaCO_3_ shell and surface-modified with PEG to create DMXAA@CBTNps. Experimental results confirmed that DMXAA@CBTNps release drugs around the tumor, simultaneously increasing local temperature and releasing CO_2_, thereby achieving the goals of occluding tumor blood vessels and reducing tumor oxygen supply (Zeng et al., 2025).

## 5 Perspectives and conclusion

This review has elucidated the targeted strategies mediated by NPs and their applications in liver disease treatment. In contrast to the previous reviews of NPs technologies for liver targeting, the present review adopts a broader and more systematic approach. First, we provide a detailed synthesis of the liver’s unique anatomy and the principles governing the biological distribution of nanomaterials within it, serving as a foundational framework. Second, we extend the discussion beyond lipidic systems to include a side-by-side analysis of diverse nanomaterial classes, such as polymeric nanoparticles, metallic nanoparticles, and nucleic acid-based nanostructures, comparing their properties, applications, and targeting efficiencies. Finally, we systematically catalog their advanced applications across a spectrum of liver diseases, including ALF, NAFLD, liver fibrosis, and HCC, emphasizing the transformative potential of nanotechnology in advancing liver disease management.

The ability to tailor NP properties for enhanced hepatic uptake holds promise for improving the efficacy of treatments for liver diseases such as hepatitis, fibrosis, and cancer. However, several challenges must be addressed to fully harness the potential of NPs in this context. The size of NPs is a primary determinant of their biodistribution and cellular uptake. Development of precise synthesis methods that yield monodisperse NPs populations could enhance consistency and predictability of hepatic uptake (Bocca et al., 2020). Additionally, real-time monitoring techniques will aid in fine-tuning NPs size distributions for specific applications. Advanced fabrication techniques, such as those used in semiconductor manufacturing, could enable the production of well-defined shapes at the nanoscale (Malik et al., 2023). Despite passive and active targeting strategies, achieving high enough specificity to diseased hepatic cells remains a significant challenge. Concerns regarding the biodegradation pathways, chronic toxicity, and eventual clearance of NPs and their components, especially for inorganic materials, need to be thoroughly addressed. Exploring shape-dependent effects systematically will provide deeper insights into optimizing hepatic delivery. Innovative surface modifications using zwitterionic polymers or hydrophilic coatings might balance between enhancing uptake and minimizing toxicity (Mu et al., 2023).

Advancements in continuous flow reactors and automated synthesis platforms could improve scalability and reproducibility. Implementing rigorous quality control measures during production will ensure consistent performance across different batches (Zhang Q. et al., 2023). Furthermore, exploring biomimetic approaches where NP surfaces mimic natural ligands can improve specificity and reduce off-target effects. Developing more sophisticated *in vitro* liver models, such as organoids or microfluidic devices, could bridge the gap between simple cell cultures and whole organisms (Hao et al., 2020). These models would allow for high-throughput screening and better prediction of clinical outcomes.

Another significant challenge is the biocompatibility and long-term toxicity of NPs. While many NP formulations have shown efficacy in pre-clinical models, concerns persist regarding their potential to induce immune responses or accumulate in off-target organs over time (Stalder et al., 2022). Development of novel ligands with higher affinity and specificity for markers uniquely overexpressed in specific liver diseases and cell types. Future studies must prioritize rigorous safety assessments to ensure that NPs can be used safely in clinical settings.

Looking ahead, the integration of multifunctional NPs that combine targeting ligands, therapeutic agents, and imaging probes holds great promise for theranostic applications. Such systems could enable real-time monitoring of treatment response and facilitate adaptive therapies tailored to evolving disease states. Additionally, exploring novel NP materials and surface coatings that enhance stability, reduce immunogenicity, and improve targeting specificity will be pivotal areas of research.

In conclusion, while NPs present a transformative opportunity in liver disease management, overcoming the aforementioned challenges through interdisciplinary collaboration and innovative research methodologies will be key to unlocking their full therapeutic potential and bringing them from bench to bedside.

## Abbreviations

ALF, acute liver failure; AM, amlodipine; ApoE, apolipoprotein E; ASGPR, asialoglycoprotein receptor; AuNPs, gold nanoparticles; CBT, calcium-based thermal-sensitive enhancer; CK, ginsenoside compound K; DCR3, decoy receptor 3; EGFR-2, endothelial growth factor receptor-2; GalNAc, N-acetylgalactosamine; HCC, hepatocellular carcinoma; HER2, epidermal growth factor receptor-2; HSCM, hepatic stellate cell membranes; HSCs, hepatic stellate cells; IFN-γ, interferon-gamma; IL-2, interleukin-2; IL-4, interleukin-4; IL-10, interleukin-10; KCs, Kupffer cells; KMD, ketalized maltodextrin; LNP, Lipid-based NPs; LPS, lipopolyscharide; LSECs, sinusoidal endothelial cells; MPS, mononuclear phagocyte system; NAFLD, non-alcoholic fatty liver disease; NP, nanoparticles; PEG, polyethylene glycol; ROS, reactive oxygen species; SR-A, scavenger receptor-A.
